# The Importance of Resolvin D1, LXA4, and LTB4 in Patients with Acute Pancreatitis Due to Gallstones

**DOI:** 10.3390/medicina61020239

**Published:** 2025-01-29

**Authors:** Naile Fevziye Mısırlıoglu, Sefa Ergun, Suat Hayri Kucuk, Solen Himmetoglu, Gulenay Defne Ozen, Ugurcan Sayili, Nedim Uzun, Hafize Uzun

**Affiliations:** 1 Department of Biochemistry, Gaziosmanpaşa Training and Research Hospital, University of Health Sciences, 34255 Istanbul, Turkey; 2 Department of General Surgery, Cerrahpasa Faculty of Medicine, Istanbul University-Cerrahpasa, 34098 Istanbul, Turkey; sefaergn@yahoo.com; 3 Department of Biochemistry, Istanbul Physical Medicine and Rehabilitation Training and Research Hospital, University of Health Sciences, 34186 Istanbul, Turkey; suatkucuk@gmail.com; 4 Department of Medical Biochemistry, Faculty of Medicine, Biruni University, 34015 Istanbul, Turkey; shimmetoglu@biruni.edu.tr; 5 Biruni University Research Center (B@MER), Biruni University, 34015 Istanbul, Turkey; 6 McGill University, Montreal, QC H3A 0G4, Canada; daphne.oz@mail.mcgill.ca; 7 Department of Public Health, Cerrahpasa Faculty of Medicine, Istanbul University-Cerrahpasa, 34098 Istanbul, Turkey; ugurcan.sayili@iuc.edu.tr; 8 Department of Emergency, Gaziosmanpaşa Training and Research Hospital, University of Health Sciences, 34255 Istanbul, Turkey; nedim.uzun@sbu.edu.tr; 9 Department of Medical Biochemistry, Faculty of Medicine, Istanbul Atlas University, 34403 Istanbul, Turkey; huzun59@hotmail.com

**Keywords:** acute pancreatitis, cholecystitis, leukotriene B4, lipoxin A4, resolvin D1

## Abstract

*Background and Objectives:* Acute pancreatitis (AP) is an inflammatory disease where there is autodigestion of the pancreas by prematurely activated enzymes which may lead to a systemic inflammatory response. The aim of our study was to investigate the levels of circulating serum leukotriene B4 (LTB4), lipoxin A4 (LXA4), and resolvin D1 (RvD1) in pancreatitis due to gallstones in the etiologic investigation of AP. *Materials and Methods:* A total of 147 patients with AP (*n*: 49), AC (*n*: 49), and combined AP + AC (*n*: 49) will be included in the study. Healthy volunteers (*n*: 49) will be included as the control group. *Results:* RvD1 levels were significantly lower in patient groups compared to controls, while LXA4 levels were lower in patients with combined AP + AC (145.24 ng/L) compared to both controls (312.36 ng/L) and other patient groups. LTB4 levels were elevated in all patient groups compared to controls (335.56 ng/L vs. 65.56 ng/L) and were highest in combined AP + AC. Significant correlations were identified: RvD1 showed a negative correlation with LTB4 (r =−0.676; *p* < 0.001) and a positive correlation with LXA4 (r = 0.563, *p* < 0.001). ROC analysis demonstrated high diagnostic accuracy, with LXA4 and LTB4 achieving perfect differentiation (AUC: 1.0) between control and combined AP + AC cases. *Conclusions:* Our study showed that serum RvD1 and LXA4 levels have powerful anti-inflammatory properties in accordance with the literature. LTB4 may represent new, effective indicators to predict the severity of AP and the presence of necrosis in patients with AP. Despite its low sensitivity and specificity, RvD1 could be used as a complementary marker to the current scoring systems for the initial assessment of AP prognosis. These findings provide a new mechanistic understanding of how RvD1 attenuates inflammation to facilitate resolution, which could help develop novel therapeutic strategies for diseases caused by unresolved inflammation. It is easily obtainable and can provide additional prognostic information to clinicians.

## 1. Introduction

Acute pancreatitis (AP) is an important disease with no specific treatment and high mortality. A major problem in the development of definitive treatments is the difficulty in understanding the pathogenesis of the disorder. In recent years, significant progress has been made in elucidating the mechanisms of the key processes underlying this disorder. A better understanding of the roles of these mechanisms may lead to the development of new therapeutic strategies [1]. Multiple etiologic factors are involved in the pathogenesis of AP. Passage of gallstones through the ampulla is the most common cause of AP. Although gallstones are responsible for 35% of AP, only 3–5% of patients with gallstones develop AP [2]. Cholecystectomy and removal of choledochal stones before AP develops reduce the risk of developing pancreatitis.

Imbalances between proinflammatory and proresolving mediators can lead to chronic inflammatory diseases. The balance of arachidonic acid-derived mediators in leukocytes is thought to be maintained through the intracellular localization of 5-lipoxygenase (5-LOX): nuclear 5-LOX promotes the biosynthesis of proinflammatory leukotriene B4 (LTB4), whereas theoretically cytoplasmic 5-LOX promotes the biosynthesis of solvent lipoxin A4 (LXA4) [3,4]. Experimental studies have shown that LXA4 can alleviate symptoms of AP-associated acute lung injury (ALI) [5,6]. In another experimental severe acute pancreatitis (SAP), LXA4 showed protective effects by inhibiting the nuclear factor-kappa B (NF-κB) signaling pathway, reducing the production of proinflammatory cytokines [7]. The LXA4 receptor agonist BML-111 also shows therapeutic potential for AP–intestinal injury in vivo and vitro [8].

Specialized proresolution mediators such as resolvin D1 (RvD1) are endogenous molecules that both reduce inflammation and promote tissue resolution without compromising host defenses. By providing a novel mechanistic understanding of how RvD1 attenuates inflammation to facilitate resolution, these findings may help to develop novel therapeutic strategies for diseases caused by unresolved inflammation [9]. This balance is shifted in favor of LXA4 by RvD1, a specialized proresolving mediator derived from docosahexaenoic acid, but the mechanism is unknown. A novel pathway has been reported in which RvD1 promotes nuclear exclusion of 5-LOX and thus suppresses LTB4 and increases LXA4 in macrophages. These findings may help develop novel therapeutic strategies for diseases caused by unresolved inflammation by providing a new mechanistic understanding of how RvD1 attenuates inflammation to facilitate resolution. RvD1 suppresses cytosolic calcium by activating its receptor, the formyl peptide receptor2/lipoxin A4 receptor, and reduces activation of the calcium-sensitive kinase calcium–calmodulin-dependent protein kinase II [10,11,12,13]. RvD1 reduced disease severity in experimental models of AP [14,15,16,17,18].

Knowledge of the above mechanisms may provide new strategies to promote inflammation resolution in chronic inflammatory diseases such as pancreatitis. The aim of our study was to investigate the levels of circulating LTB4, LXA4, and resolvin D1 in pancreatitis due to gallstones in the etiologic investigation of AP, which are among the causes of serious morbidity and mortality.

## 2. Materials and Methods

### 2.1. Subjects

This study was conducted according to the guidelines of the Declaration of Helsinki and approved by the Istanbul University-Cerrahpasa, Cerrahpasa Medical Faculty Clinical Research Ethics Committee (approval number: 1108507; date: 3 October 2024). All subjects gave their informed consent for inclusion before they participated in the study. All subjects were of Turkish descent.

### 2.2. Sample Size and Study Groups

The sample size was determined using G*Power 3.1 software. Since no previous studies were available examining LTB4, LXA4, and resolvin D1 in combined pancreatitis and cholecystitis patients, we performed a power analysis with the following parameters: α error probability of 0.05, power (1-β) of 0.80, 4 groups, and a medium effect size (f = 0.25). This analysis indicated a minimum required sample size of 180 participants (45 per group). To account for potential dropouts and increase statistical power, we included 49 participants in each group.

A total of 147 patients with AP and cholecystitis from Gaziosmanpasa Training and Research Hospital, Emergency Department, were included in the study. In the control group who were age- and gender-matched with the patients, 49 individuals who were admitted to the check-up center of our hospital for routine controls and who did not have chronic diseases and active infections were included.

The AP group consisted of patients who presented to our clinic with abdominal pain. AP was diagnosed according to the Revised Atlanta Criteria (2012) [19]. Patients with more than 3-fold higher amylase and/or lipase levels, characteristic abdominal pain, and two-thirds of the imaging findings were considered as AP and included in the study.

Acute cholecystitis (AC) was diagnosed as recommended in the Tokyo guidelines by clinical examination and confirmed by ultrasound scan [20].

### 2.3. Inclusion Criteria

(i) Patients followed with a prediagnosis of AP; (ii) patients followed with a prediagnosis of AC; (iii) patients with concurrent cholecystitis and gallstone pancreatitis, and (iv) patients and healthy volunteers who accepted and signed the informed consent form were included in the study.

### 2.4. Exlusion Criteria

Patients under 20 years of age, pregnant women, patients with metabolic diseases, patients with inflammatory diseases, patients with oncology and hematologic diseases, patients receiving steroid and antibiotic treatment during hospitalization, patients with multitrauma, patients who did not want to participate voluntarily, and patients with incomplete data were excluded from the study.

### 2.5. Sample Collection and Measurements

Venous blood samples were taken from the subjects for analysis 6 h after the onset of pain (this is the onset of AP). Blood samples were drawn from the brachial veins in brachial fossa and placed into plain tubes and anticoagulant-free tubes. The samples were centrifuged for 10 min at 4000 rpm at 4 °C. Biochemical tests were performed immediately. For the determination of other parameters, serum aliquots were frozen and stored at –80 °C immediately until they were required for further analysis.

### 2.6. Measurement of Serum Resolvin D1 (RvD1) Levels

Serum RvD1 sandwich ELISA kit was used according to the manufacturer’s instructions (Human Leukotriene B4 ELISA Kit, Catalog No: E7450Hu, Bioaasay Technology Laboratory (BT LAB), Zhejiang, China). All samples were examined twice. The minimum measurable level for serum LTB4 was 19.01 ng/mL. Intra- and inter-CV for LTB4 were determined to be 8% and 9%, respectively.

### 2.7. Measurement of Serum Lipoxin A4 (LXA4) Levels

Serum LXA4 competitive ELISA Kit was used according to the manufacturer’s instructions (Human Lipoxin A4 ELISA Kit, Catalog No: E-EL-0053, Elabscience, Houston, TX, USA). All samples were examined twice. The minimum measurable level for serum LTB4 was 0.47 ng/mL. Intra- and inter-CV for LTB4 were determined to be 8% and 10%, respectively.

### 2.8. Measurement of Serum Leukotriene B4 (LTB4) Levels

Serum LTB4 sandwich Enzyme-Linked Immuno Sorbent Assay (ELISA) kit was used according to the manufacturer’s instructions (Human Leukotriene B4 ELISA Kit, Catalog No: E1540Hu, Bioaasay Technology Laboratory (BT LAB), Zhejiang, China). All samples were examined twice. The minimum measurable level for serum LTB4 was 1.09 ng/mL. Intra- and inter-CV for LTB4 were determined to be 9% and 11%, respectively.

Biochemical parameters were determined using the enzymatic methods (COBAS 8000, ROCHE-2007, Tokyo, Japan).

### 2.9. Statistical Analysis

Statistical analyses were conducted using SPSS software version 21.0 (IBM Corp., Armonk, NY, USA). JASP 0.16.4.0 and Jamovi 2.3.18 were used to generate graphs. Categorical variables are presented as frequencies and percentages, while continuous variables are presented as mean ± SD or median and interquartile range (IQR) values. The normality of data distribution was assessed using the Shapiro–Wilk test. For comparison of categorical variables across groups, the chi-square test was applied. According to the normality of distribution, continuous variables were compared across groups using either the Kruskal–Wallis test or one-way ANOVA. When a significant difference was identified, post hoc analysis was conducted using Tukey’s test or adjusted *p*-values to determine the source of the differences. Receiver operating characteristic (ROC) curve analysis was performed to evaluate the diagnostic performance of RvD1, LXA4, and LTB4, with the area under the curve (AUC), sensitivity, specificity, and optimal cutoff values reported. Cut-off values were determined using the Youden index (J = Sensitivity + Specificity − 1) and subsequently rounded to the nearest practical value to enhance clinical utility while maintaining optimal diagnostic performance. A *p*-value < 0.05 was considered statistically significant.

## 3. Results

The demographic and clinical characteristics of the study groups, including control, AC, AP, and AP combined with cholecystitis, are presented in Table 1. Gender distribution and the mean age did not show significant differences among the groups (*p* = 0.338, 0.659). However, the body mass index (BMI) was significantly higher in the patient groups (AC, AP, and combined cases) compared to the control group (*p* < 0.001). Also, fasting blood glucose (FBG) was significantly higher in AP and combined AP + AC groups compared to control and AC (*p* < 0.001). Diabetes and hypertension rates of AP and combined C groups are greater than control and AC. Amylase and lipase levels were significantly higher in the AP and combined AP + AC groups compared to the control and AC groups (*p* < 0.001). Total cholesterol and LDL cholesterol levels were significantly higher in all patient groups compared to the control group, while HDL cholesterol was significantly lower (*p* < 0.001). Triglyceride levels were significantly elevated in the AC and AP groups compared to the control group (*p* = 0.003). AST and ALT levels were significantly higher in the combined AP + AC groups compared to the other groups (*p* < 0.001). LDH was significantly higher in AC than the other groups (*p* < 0.001). WBC and CRP levels were significantly elevated in all patient groups compared to the control group (*p* < 0.001) (Table 1).

RvD1 levels were significantly lower in the all-patient group compared to the control group (1249.26 ng/L [976.95–1549.26]; *p* < 0.001). Also, RvD1 levels were significantly lower in the combined AP + AC group (238.45 ng/L [205.56–313.65]) than AC (316.42 ng/L [228.45–416.12]).

**Figure 1:**
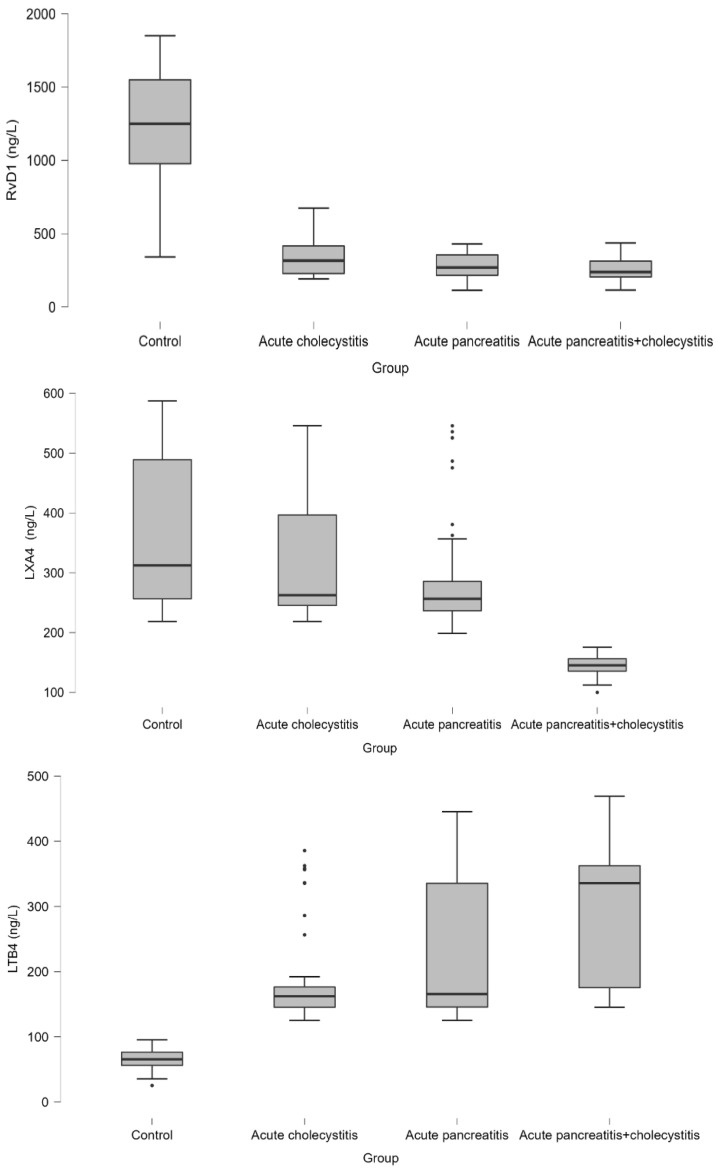
The box-plot graphs for resolvin D1, LXA4, and LTB4 by control and patient groups.

LXA4 levels were significantly lower in the combined AP + AC groups (145.24 ng/L [136.56–156.45]) compared to the other groups (AP (256.45 ng/L [236.42–285.56]), AC (262.45 ng/L [245.24–396.56]), and the control group (312.36 ng/L [256.45–489]; *p* < 0.001)). LTB4 levels were significantly higher in the combined AP + AC groups (335.56 ng/L [175.56–362.45]) compared to the other groups. Also, it was higher in the AC (162.45 ng/L [145.24–176.42]), AP (165.65 ng/L [145.65–335.56]), and control group (65.56 ng/L [56.23–76.42]; *p* < 0.001) (Table 1, Figure 1).

**Figure 2:**
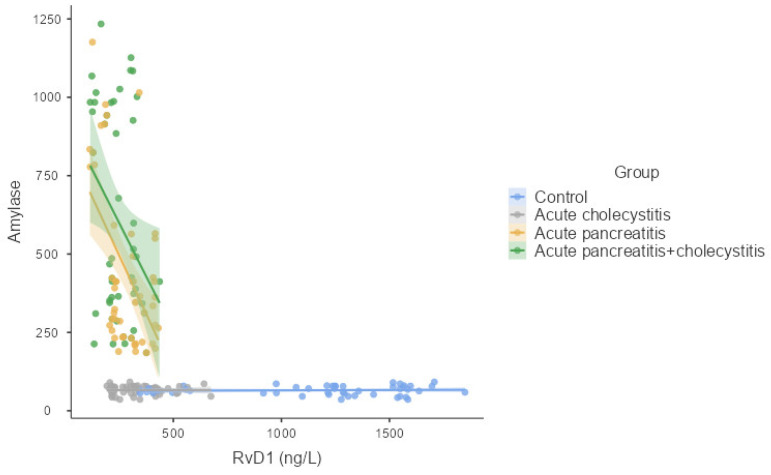

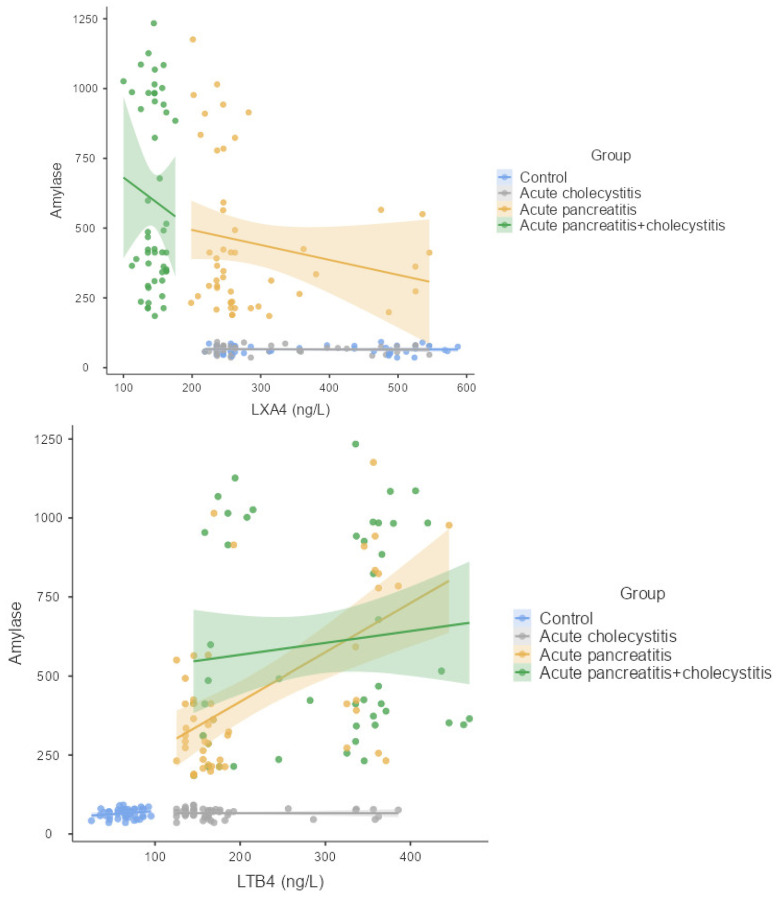
Correlation of RvD1, LTA4, and LXB4 with amylase.

Significant correlations among RvD1, LXA4, and LTB4 were identified in Table 2. A positive correlation was found between RvD1 and LXA4 (r = 0.563, *p* < 0.001). In contrast, a negative and significant correlation was observed between RvD1 and LTB4 (r = −0.676, *p* < 0.001) and between LXA4 and LTB4 (r = −0.518, *p* < 0.001). As shown in Table 2, RvD1, LXA4, and LTB4 levels exhibited distinct correlations with various biochemical and demographic variables. RvD1 levels had a significant negative correlation with amylase (r = −0.564, *p* < 0.001) and lipase (r = −0.623, *p* < 0.001), indicating an inverse relationship with pancreatic enzyme activity. Additionally, RvD1 showed a negative correlation with CRP (r = −0.637 *p* < 0.001), reflecting its association with the inflammatory response. LXA4 levels demonstrated a negative correlation with amylase (r = −0.554, *p* < 0.001) and lipase (r = −0.544, *p* < 0.001), indicating a moderate association with pancreatic enzymes. Furthermore, LXA4 showed a negative correlation with CRP (r = −0.323, *p* < 0.001), underscoring its association with inflammation levels. LTB4 also exhibited significant positive correlations with amylase (r = 0.609, *p* < 0.001) and lipase (r = 0.608, *p* < 0.001), highlighting its association in inflammatory processes. Additionally, LTB4 showed a positive correlation with CRP (r = 0.660, *p* < 0.001), further supporting its role in the inflammatory response (Table 2, Figure 2).

The ROC analysis results for RvD1, LXA4, and LTB4 are presented in Table 3, evaluating their diagnostic performance in differentiating between control and patient groups. For distinguishing control from combined AP + AC, RvD1 achieved an area under the curve (AUC) of 0.995 (95% CI: 0.987–1.0, *p* < 0.001), with a sensitivity of 95.9% and specificity of 95.9% at a cutoff of lower than 375 ng/L.

LXA4 had a significant AUC of 1.0 (95% CI: 1.0–1.0, *p* < 0.001) for distinguishing control from combined AP + AC, with a sensitivity of 100% and specificity of 100% at a cutoff of lower than 200 ng/L. LTB4 had a significant AUC of 1.0 (95% CI: 1.0–1.0, *p* < 0.001) for distinguishing control from combined AP + AC, with a sensitivity of 100% and specificity of 100% at a cutoff of 125 ng/L.

For differentiating AC from combined AP + AC, RvD1 had an AUC of 0.717 (95% CI: 0.617–0.817, *p* < 0.001), with a sensitivity of 91.8% and specificity of 44.9% at a cutoff of 333 ng/L. LXA4 had a significant AUC of 1.0 (95% CI: 1.0–1.0, *p* < 0.001) for distinguishing AC from combined AP + AC, with a sensitivity of 100% and specificity of 100% at a cutoff of lower than 200 ng/L. LTB4 had a significant AUC of 0.815 (95% CI: 0.732–0.898, *p* < 0.001) for distinguishing AC from combined AP + AC, with a sensitivity of 73.5% and specificity of 79.6% at a cutoff of 185 ng/L.

For differentiating AP from combined AP + AC, RvD1 did not show significant AUCs. LXA4 had a significant AUC of 1.0 (95% CI: 1.0–1.0, *p* < 0.001) for distinguishing AP from combined AP + AC, with a sensitivity of 100% and specificity of 100% at a cutoff of lower than 195 ng/L and with a sensitivity of 100% and specificity of 98% at a cutoff of lower than 200 ng/L. LTB4 had a significant AUC of 0.717 (95% CI: 0.616–0.818, *p* < 0.001) for distinguishing AP from combined AP + AC, with a sensitivity of 73.5% and specificity of 65.3% at a cutoff of 185 ng/L (Table 3).

## 4. Discussion

Acute pancreatitis (AP) is a local and systemic inflammation caused by activation of pancreatic enzymes in the gland parenchyma for various etiologic reasons. The disease has serious socioeconomic consequences due to its increasing global incidence, prolonged hospitalizations, and long-term endocrine and exocrine pancreatic insufficiency. In the current study, we aimed to identify new markers that guide the diagnosis of the disease and determine its severity. Gallbladder stones and alcohol constitute most of the etiology of AP [21]. The most common etiologic cause in our country is gallstones. There are no studies in the literature examining the association of RvD1, LXA4, and LTB4 in AP due to gallstones, but there are many studies examining the association of these three molecules with other inflammatory diseases. In the current study, serum RvD1 and LXA4 levels were significantly lower in the all-patient group compared to the control group. RvD1 and LXA4 levels were also significantly lower in the combined AP + AC group than AC. LTB4 levels were significantly higher in the AC, AP, and combined AP + AC groups compared to the control group. A positive correlation was found between RvD1 and LXA4, while a negative correlation was found with LTB4. A negative and significant correlation was observed between LXA4 and LTB4. Decreased serum RvD1 and LXA4 and increased serum LXB4 levels can be used as a useful biomarker to differentiate active patients from healthy individuals in disease diagnosis. In addition, RvD1 and LXA4 levels were lower in the group with severe disease, which will guide clinicians in determining the prognosis of the disease. Our study showed that serum RvD1 and LXA4 levels have powerful anti-inflammatory properties in accordance with the literature. LTB4 may represent new, effective indicators to predict the severity of AP and presence of necrosis in patients with AP.

RvD1, involved in resolution, maintains homeostasis of acute inflammation by decreasing neutrophil migration and increasing macrophage efferocytosis. RvD1 is a potent stereoselective agonist that controls the duration and magnitude of inflammation [22]. In the current study, RvD1 levels were found to be different between AP and control group, AC and control group, and combined AP + AC and AC groups. RvD1 levels differ between groups with AP, AC, and controls. RvD1 showed a strong negative correlation with amylase levels. These results suggest that RvD1 may be involved in anti-inflammatory processes in these conditions. Especially in inflammation-related diseases such as AP, AC, and combined AP + AC, RvD1 has the potential to resolve inflammation and reduce tissue damage. In the experimental models, RvD1 has been reported to have a protective role for AP, chronic pancreatitis (CP), and diabetes mellitus (DM) [15,16,17,23,24]. According to the pancreatic injury scoring system established by Schimdt et al. [25], while edematous expansion of the intra-acinar septa was observed in the AP group due to pancreatitis, edematous expansion of the intralobular septa and preservation of the intra-acinar septa were observed in rats with AP treated with RvD1, indicating resolution of edema. The key observations indicate that RvD1 might have a protective and potentially therapeutic role in reducing pancreatic injury and inflammation, which is significant because the pathogenesis of AP is complex and not fully understood. The most important problem in developing definitive treatments for AP is the difficulty in understanding the pathogenesis of the disorder. In the future, RvD1 may also reduce the severity of severe AP in humans, as shown in experimental studies.

Although gallstones are responsible for 35% of AP, only 3–5% of patients with gallstones develop AP [2]. Cholecystectomy and choledochal stone removal procedures performed before the development of AP reduce the risk of pancreatitis. The relationship between bile sludge and pancreatitis has not been proven. Although there are no prospective randomized studies, some uncontrolled studies have shown that cholecystectomies performed in patients with pancreatitis with bile sludge are beneficial in preventing subsequent attacks [26,27]. The mortality rate in biliary pancreatitis is around 8% in the first attack and 1% in subsequent attacks [20]. In the current study, for distinguishing control from AP combined with AC, RvD1 achieved this with a sensitivity of 95.9% and specificity of 95.9% at a cutoff of lower than 375 ng/L. For differentiating AC from APC, RvD1 had a sensitivity of 91.8% and specificity of 44.9% at a cutoff of 333 ng/L. RvD1 demonstrated high diagnostic performance in distinguishing AP combined with cholecystitis from the control group, achieving high sensitivity and high specificity. For differentiating AC from AP with APC, RvD1 showed high sensitivity and low specificity. Serum RvD1 may be useful in preventing subsequent attacks of cholecystectomies performed in patients with pancreatitis with bile sludge.

Further, RvD1 exerts a protective effect by reducing neutrophil migration in tissue damage secondary to ischemia–reperfusion. RvD1 levels tend to increase secondary to inflammation because of its neuroprotective and anti-inflammatory properties. Rvs show great anti-inflammatory effects in neurodegenerative diseases, neuroimmune diseases, and cerebrovascular disorders, thus providing a new way of thinking for the treatment and prevention of neurological diseases [28].

Lipoxins biosynthesized from arachidonic acid are potent, active stop signals for neutrophils (polymorphonuclear leukocyte (PMN) infiltration [29,30]) and are produced during resolution of self-limited inflammatory responses [31,32]. In the current study, LXA4 levels were significantly lower in the AC, AP, and combined pancreatitis–cholecystitis groups compared to the control group. LTB4 levels were significantly higher in combined pancreatitis–cholecystitis groups compared to the other groups and were higher in the AC and AP groups than the control group. In fact, the levels of leukotriene B4 (LTB4), 15 hydroxyeicosatetraenoic acid (15-HETE), thromboxane B2 (TXB2), and prostaglandin E2 (PGE2) increased in pancreatic tissue upon the induction of AP [33,34]. Shahid et al. [35] found that administration of caerulein or intraductal bile acids in mice causes production of LTB_4_ by pancreatic acinar cells. The administration of LTB4 induced pancreatic damage, evidenced by pancreatic edema, neutrophil infiltration, and necrosis. Common pancreaticobiliary duct obstruction causes an increase in pancreatic LTB4 levels that in turn mediates activation of the transient receptor potential vanilloid 1 (TRPV1), resulting in AP. The administration of LTB4 induced pancreatic damage, evidenced by pancreatic edema, neutrophil infiltration, and necrosis [36]. LXA4, a potent available anti-inflammatory and novel antioxidant mediator, has been extensively studied in AP-associated acute lung injury (ALI). However, the mechanism of protection has not yet been entirely elucidated [37]. Ye et al. [5] showed that mice pretreated with LXA4 exhibited obviously reduced serum amylase levels and lung Wet-to-Dry Weight (W/D) ratios and improved histopathological inflammatory damage in the lungs and pancreas. These results suggested that LXA4 can ameliorate the severity of AP and ALI. In addition, in severe AP-induced ALI, LXA4 attenuates adhesion and exerts cytoprotection by preserving mitochondrial function [5]. Intramuscular diclofenac after endoscopic retrograde cholangiopancreatography (ERCP) in 30 patients from the diclofenac group and 30 patients from the control group can reduce the incidence of pancreatitis. This may be related to the fact that diclofenac can increase the levels of LxA4, RvD1, and RvE1 [18]. LXA4 may exert its protective effects against AP by inhibiting the inflammatory pathway LTB4 and exerting a cytoprotective effect through interaction with RvD1. Our results confirmed that LXA4 provides protection in AP through its anti-inflammatory effect. In the literature, there are new biomarkers like butyrylcholinesterase (BChE) that are used as a marker for the risk of developing surgical site infection (SSI) and septic complications in patients undergoing colorectal surgery. Verras et al. [38] showed that low levels of BChE in the first and third postsurgery were associated with an increased risk for the development of SSIs but not sepsis.

### The Study’s Limitations

Since the inflammatory process was investigated in our study, careful selection of the patient group and investigation of LXs and resolvins, which are important in the resolution of inflammation, are the strengths of our study. However, the small number of patients included in the study and the absence of patients with other etiologies of pancreatitis are important limitations of the study. In light of all these data, large population studies in which procalcitonin, inflammatory cytokines, and ASA score can be investigated are needed to determine the role of RvD1, LXA4, and LTB4 in the etiopathogenesis of AP.

## 5. Conclusions

RvD1, LXA4, and LTB4 play key roles in the control and resolution of inflammation. RvD1 and LXA4 have been implicated as possible anti-inflammatories, and their pro-resolution bioeffects have been reinforced for years. The effects of these molecules have been reported in a wide range of animal studies and in vitro models of disease and continue to guide human therapeutics. The inflammation-resolving process of RvD1 and LXA4 prevents tissue injury and inhibits the transition of acute inflammation to chronic inflammation. RvD1, which acts as an anti-inflammatory agent, may activate LXA4 while blocking or inhibiting both exogenous and endogenous proinflammatory mediators such as LTB4. These findings provide a new mechanistic understanding of how RvD1 attenuates inflammation to facilitate resolution, which could help develop novel therapeutic strategies for diseases caused by unresolved inflammation. RvD1 and LXA4 levels were lower in the group with severe disease, which will guide clinicians in determining the prognosis of the disease.

## Figures and Tables

**Table 1:** Demographic and clinical characteristics of study groups.

	Control	Acute Cholecystitis	Acute Pancreatitis	Acute Pancreatitis+ Acute Cholecystitis	*p*-Value
**Gender (Female)**	27 (55%)	27 (55%)	31 (63%)	26 (53%)	0.338 ¶
**Age (Year)**	64.98 ± 11.32	66.2 ± 10.42	63.67 ± 11.83	63.71 ± 12.43	0.659 †
**NAFLD** (*n*, %)	0 (0%) ^a^	0 (0%) ^a^	25 (51%) ^b^	29 (59.2%) ^b^	<0.001 ^Ω^
**DM** (*n*, %)	0 (0%) ^a^	0 (0%) ^a^	43 (87.8%) ^b^	34 (69.4%) ^b^	<0.001 ^Ω^
**HT** (*n*, %)	0 (0%) ^a^	13 (26.5%) ^b^	21 (42.9%) ^b^	25 (51%) ^b^	<0.001 ^Ω^
**BMI (kg/m^2^)**	24.01 ± 2.11 ^a^	26.66 ± 4.04 ^b^	26.98 ± 4.31 ^b^	26.83 ± 3.75 ^b^	<0.001 †
**FBG (mg/dL)**	80.02 ± 9.75 ^a^	88.01 ± 6.49 ^a^	144.53 ± 42.63 ^b^	169.04 ± 76.45 ^c^	<0.001 †
**Amylase (U/L)**	68 (56–78) ^a^	70 (57–76) ^a^	346 (236–564) ^b^	425 (345–954) ^b^	<0.001 *
**Lipase (U/L)**	34.58 (23–48.54) ^a^	42 (23.65–48.54) ^a^	102.56 (86–145) ^b^	132 (86.45–213.9) ^b^	<0.001 *
**T.Cholesterol (mg/dL)**	168.2 (159.8–180.4) ^a^	213 (203–220.4) ^b^	213 (203–219.6) ^b^	212.4 (199–220.4) ^b^	<0.001 *
**LDL (mg/dL)**	103 (94–109) ^a^	145 (137–154) ^b^	146 (138–153) ^b^	146 (137–153) ^b^	<0.001 *
**HDL (mg/dL)**	46 (43–51) ^a^	38 (36–46) ^b^	42 (34–48) ^b^	41 (36–45) ^b^	<0.001 *
**Triglyceride (mg/dL)**	110 (97–115) ^a^	120 (110–180) ^b^	115 (108–150) ^b^	112 (93–135) ^a,b^	0.003 *
**AST (U/L)**	23 (16–26) ^a^	20.3 (16–23)^a^	23 (16–55) ^a^	145 (102–302) ^b^	<0.001 *
**ALT (U/L)**	24.1 (18.4–33) ^a^	23.1 (18.5–31 ^a^	34 (22–47.4) ^a^	122 (76–206) ^b^	<0.001 *
**LDH (U/L)**	98 (86–134) ^a^	177 (155–203) ^c^	123 (92–162) ^a,b^	124 (96–268) ^b^	<0.001 *
**WBC (10^3^/µL)**	8.1 (6.8–8.9) ^a^	10.2 (8.6–14.3) ^b^	10.2 (8.4–14.5) ^b^	13.1 (9.7–17.5) ^b^	<0.001 *
**CRP (mg/L)**	2.7 (2.1–4) ^a^	58.6 (46.5–78.6) ^b^	58.6 (46.1–78.1) ^b^	59.1 (46.5–132.5) ^b^	<0.001 *
**RvD1 (ng/L)**	1249.26 (976.95–1549.26) ^a^	316.42 (228.45–416.12) ^b^	269.24 (216.45–356.12) ^b,c^	238.45 (205.56–313.65) ^c^	<0.001 *
**LXA4 (ng/L)**	312.36 (256.45–489) ^a^	262.45 (245.24–396.56) ^a^	256.45 (236.42–285.56) ^a^	145.24 (135.56–156.45) ^b^	<0.001 *
**LTB4 (ng/L)**	65.56 (56.23–76.42) ^a^	162.45 (145.24–176.42) ^b^	165.65 (145.65–335.56) ^b^	335.56 (175.56–362.45) ^c^	<0.001 *

¶: chi-square test; ^Ω^: Fisher’s exact test; †: one-way ANOVA; *: Kruskal–Wallis test were applied. Different superscript letters indicate groups with significant differences.

**Table 2:** Correlation analysis of RvD1, LXA4, and LTB4 with biochemical and demographic variables.

		All Groups			Control			Acute Cholecystitis			Acute Pancreatitis			Acute Pancreatitis + Cholecystitis		
Variable		RvD1 (ng/L)	LXA4 (ng/L)	LTB4 (ng/L)	RvD1 (ng/L)	LXA4 (ng/L)	LTB4 (ng/L)	RvD1 (ng/L)	LXA4 (ng/L)	LTB4 (ng/L)	RvD1 (ng/L)	LXA4 (ng/L)	LTB4 (ng/L)	RvD1 (ng/L)	LXA4 (ng/L)	LTB4 (ng/L)
**RvD1 (ng/L)**	**r**	1.000	0.563 **	−0.676 **	1.000	0.383 **	0.086	1.000	0.338 *	0.202	1.000	0.717 **	−0.712 **	1.000	0.014	0.010
	***p***		<0.001	<0.001		0.007	0.556		0.018	0.165		<0.001	<0.001	<0.001	0.924	0.947
**LXA4 (ng/L)**	**r**	0.563 **	1.000	−0.518 **	0.383 **	1.000	−0.067	0.338 *	1.000	0.330 *	0.717 **	1.000	−0.539 **	0.014	1.000	−0.005
	***p***	<0.001		<0.001	0.007		0.647	0.018		0.021	<0.001		<0.001	0.924		0.972
**LTB4 (ng/L)**	**r**	−0.676 **	−0.518 **	1.000	0.086	−0.067	1.000	0.202	0.330 *	1.000	−0.712 **	−0.539 **	1.000	0.010	−0.005	1.000
	***p***	<0.001	<0.001		0.556	0.647		0.165	0.021		<0.001	<0.001		0.947	0.972	
**Amilase (U/L)**	**r**	−0.564 **	−0.554 **	0.609 **	0.062	−0.018	0.202	−0.057	−0.011	−0.058	−0.441 **	−0.242	0.371 **	−0.256	−0.044	0.231
	***p***	<0.001	<0.001	<0.001	0.674	0.904	0.164	0.700	0.943	0.690	0.002	0.093	0.009	0.076	0.761	0.111
**Lipase (U/L)**	**r**	−0.623 **	−0.544 **	0.608 **	−0.225	−0.010	0.063	−0.218	−0.072	0.068	−0.482 **	−0.398 **	0.531 **	−0.432 **	−0.009	0.062
	***p***	<0.001	<0.001	<0.001	0.120	0.946	0.668	0.132	0.624	0.644	<0.001	0.005	<0.001	0.002	0.949	0.673
**CRP (mg/L)**	**r**	−0.637 **	−0.323 **	0.660 **	−0.023	−0.044	0.028	0.086	0.271	0.164	−0.399 **	−0.230	0.248	−0.408 **	0.024	0.106
	***p***	<0.001	<0.001	<0.001	0.875	0.764	0.849	0.559	0.060	0.259	0.004	0.112	0.086	0.004	0.870	0.467

*: *p* < 0.05; **: *p* < 0.01.

**Table 3:** ROC analysis results for diagnostic efficacy of RvD1, LXA4, and LTB4.

	Variable	AUC	95% CI	*p*-Value	Cutoff	Sensitivity	Specificity
**AC vs. combined AP + AC**	**RvD1**	0.995	0.987–1.000	<0.001	375 *	95.9%	95.9%
	**LXA4**	1	1–1	<0.001	200 *	100%	100%
	**LTB4**	1	1–1	<0.001	125 †	100%	100%
**AC vs. combined AP + AC**	**RvD1**	0.717	0.617–0.817	<0.001	333 *	91.8%	44.9%
	**LXA4**	1	1–1	<0.001	200 *	100%	100%
	**LTB4**	0.815	0.732–0.898	<0.001	185 †	73.5%	79.6%
**AP vs. combined AP + AC**	**RvD1**	0.610	0.498–0.723	0.060			
	**LXA4**	1	1–1	<0.001	195 *	100%	100%
					200	100%	98%
	**LTB4**	0.717	0.616–0.818	<0.001	185 †	73.5%	65.3%

*: lower value; †: greater value indicated by the combined AP + AC.

## Data Availability

The data underlying this article are available in the article. If needed, please contact the corresponding author. The email address is huzun59@hotmail.com.
